# Gut Microbiota-Based Algorithms in the Prediction of Metachronous Adenoma in Colorectal Cancer Patients Following Surgery

**DOI:** 10.3389/fmicb.2020.01106

**Published:** 2020-06-12

**Authors:** Yang Liu, Rui Geng, Lujia Liu, Xiangren Jin, Wei Yan, Fuya Zhao, Shuang Wang, Xiao Guo, Ghanashyam Ghimire, Yunwei Wei

**Affiliations:** Department of Oncological and Endoscopic Surgery, The First Affiliated Hospital of Harbin Medical University, Harbin, China

**Keywords:** colorectal cancer, metachronous cancer, colorectal adenoma, gut microbiota, random forest

## Abstract

Evaluating the risk of colorectal metachronous adenoma (MA), which is a precancerous lesion, is necessary for metachronous colorectal cancer (CRC) precaution among CRC patients who had underwent surgical removal of their primary tumor. Here, discovery cohort (*n* = 41) and validation cohort (*n* = 45) of CRC patients were prospectively enrolled in this study. Mucosal and fecal samples were used for gut microbiota analysis by sequencing the 16S rRNA genes. Significant reduction of microbial diversity was noted in MA (*P* < 0.001). A signature defined by decreased abundance of eight genera and increased abundance of two genera strongly correlated with MA. The microbiota-based random forest (RF) model, established utilizing *Escherichia–Shigella*, *Acinetobacter* together with BMI in combination, achieved AUC values of 0.885 and 0.832 for MA, predicting in discovery and validation cohort, respectively. The RF model was performed as well for fecal and tumor adjacent mucosal samples with an AUC of 0.835 and 0.889, respectively. Gut microbiota profile of MA still existed in post-operative cohort patients, but the RF model could not be performed well on this cohort, with an AUC of 0.61. Finally, we introduced a risk score based on *Escherichia–Shigella*, *Acinetobacter* and BMI, and synchronous-adenoma achieved AUC values of 0.94 and 0.835 in discovery and validation cohort, respectively. This study presented a comprehensive landscape of gut microbiota in MA, demonstrated that the gut microbiota-based models and scoring system achieved good ability to predict the risk for developing MA after surgical resection. Our study suggests that gut microbiota is a potential predictive biomarker for MA.

## Introduction

Colorectal cancer (CRC) is among the leading cause of cancer-related deaths worldwide. Despite substantial progress in the early diagnosis and treatment of CRC and the fact that more than two-thirds of CRC patients received surgical resection and adjuvant therapy, nearly 40% of these patients developed CRC recurrence, including local recurrence, metachronous cancer, and distant metastasis (Kahi et al., 2016). It has been well-documented that patients with a history of CRC are at an increased risk of developing metachronous CRC following surgical resection and pre-operative clearing (Balleste et al., 2007; Mulder et al., 2012). As such, post-operative colonoscopy is highly recommend for patients after surgical resection of CRC to improve survival via diagnosing metachronous CRC at an early stage or to prevent the occurrence of metachronous CRC via detecting and removing of the pre-cancerous colorectal polyps (Kahi et al., 2016). According to the major guidelines, an initial full colonoscopy is recommended at the time of diagnosis or within 3–6 months following surgical intervention for detection of synchronous lesions, while further colonoscopies should be carried out >6 months, generally 1 year after the surgical resection (Meyerhardt et al., 2013), followed by colonoscopies every 3–5 years for detection of metachronous cancer. There is no first-level evidence in support of the optimal total duration of surveillance after treatment for CRC (van der Stok et al., 2016).

Despite a certain level risk, there has been a lack of reliable factors to be used for predicting metachronous CRC in patients who have undergone surgical treatment. Thus, life-long colonoscopy surveillance is needed. Currently, several factors have been shown to be associated with an increased risk of metachronous CRC, including age, previous or synchronous adenomas or history of CRC, right-sided tumors, and microsatellite instability (MSI); many of these reported risk factors were inconsistent in the previous studies (Balleste et al., 2007; Bouvier et al., 2008). Identification of individuals at high risk for the development of metachronous colorectal cancer is necessary to increase the efficiency of surveillance and to improve prognosis.

Recent studies have suggested that the community of microbes inhabiting the gastrointestinal tract plays an important role in the development and progression of CRC (Arthur et al., 2012; Kostic et al., 2013). In fact, gut microbiota dysbiosis was already found in patients with colorectal adenoma, and the disturbance became more apparent during the progression of adenoma into CRC (Feng et al., 2015). It has been of note that gut bacteria may exert a role in tumorigenesis, and in turn, they may have potential as useful biomarkers for the early detection of disease (Zeller et al., 2014). A previous study has indicated that gut microbiota could be used to quantify the risk of recurrence (Sze et al., 2017). Until now, it remains unknown if gut microbiota could hold a value in assessment of risk for metachronous CRC or precancerous lesions such as colorectal adenoma, given the pathogenesis of CRC.

As CRC develops gradually from premalignant adenomatous, accurate prediction and early detection polyps provides an opportunity to halt this process. Our previous study found that colorectal cancer patients who developed metachronous adenoma (MA) post-operatively showed distinct fecal microbiota, which can be potentially used for diagnosis for MA (Jin et al., 2019). But, the features of MA gut microbiota that already existed before operation or formed post-operatively is still unknown. Could pre-operative gut microbiota be used as a tool to predict the risk for post-operative MA?

In this study, discovery and validation cohort of CRC patients was prospectively enrolled, the mucosal and fecal samples were used for analysis of gut microbiota by sequencing the 16S rRNA genes. We aimed to test the hypothesis that the gut microbiota composition before surgery was associated with the risk of developing MA and thus could be used, together with other independent risk factors, to generate new algorithms for better predicting MA.

## Materials and Methods

### Study Population

Colorectal cancer patients of discovery and validation cohort were both prospectively enrolled at the First Affiliated Hospital of Harbin Medical University during the period between September 2017 and April 2018. All the patients were diagnosed with primary colorectal adenocarcinoma and underwent surgical resection of CRC. During the enrollment, the patients who had the following conditions were excluded from this study: (1) taking antibiotics in 1 month prior to colonoscopy examination; (2) previous diagnosis of CRC, IBD, or IBS; and (3) medical history of surgery, radiation, or chemotherapy. A total of 41 CRC patients were in the discovery cohort, 13 patients had both fecal and colonoscopic mucosal samples, and 45 patients in the validation cohort had colonoscopic mucosal samples, but not fecal samples.

### Sample Collection

Cold biopsy forceps were used for collection of colonoscopic mucosal biopsies from CRC tissues and adjacent, cancer-free tissues (at least 5 cm away from lesions), respectively. Fecal samples were taken the night before colonoscopy examination day. All the samples were snap-frozen in cryovial immediately following collection and stored at −80°C until DNA extraction.

### Follow-Up

All the study patients were followed up for 12 months; during the follow-up period, they were scheduled to undergo surveillance colonoscopy every 1 year. Four patients in the discovery cohort and seven patients in the validation cohort, combined with a malignant bowel obstruction (MBO), were asked for colonoscopy within 2–4 months after surgery to detect synchronous lesions, followed by repeat colonoscopy at 1 year to detect metachronous lesion according to the guidelines (Meyerhardt et al., 2013). For patients with synchronous adenoma detected before surgery, endoscopic mucosa resection (EMR) was performed to remove the lesion prior to colon resection. The primary endpoint was MA detection during follow up period.

### DNA Extraction and 16S rRNA Gene Sequencing for Bacterial Identification

The fecal and mucosal samples as described in the sample collection were used for DNA extraction. In brief, microbial DNA was extracted using a DNA kit (Bio-Tek, GA, United States) according to the manufacturer’s instructions and used for an amplification of the hypervariable regions (V3-V4) of the bacterial 16S rRNA gene. The resulting amplicons were purified and pooled in equimolar concentrations, followed by paired-end sequencing (2 × 300) on an Illumina MiSeq platform (Illumina, San Diego, CA, United States), which was performed by Majorbio Bio-Pharm Technology (Shanghai, China). After the raw reads were filtered and quality control was conducted, OTUs were clustered with a 97% similarity cut-off using UPARSE^1^ (version 7.1), following which, the identified chimeric sequences were removed using UCHIME. With the RDP Classifier algorithm, taxonomic assignments for the 16S rRNA gene sequences were made^2^ with the GreenGene 16S rRNA gene database at a confidence threshold of 70%. The 16S rRNA gene sequencing runs were separately performed for the discovery and validation cohorts for both MA and nMA patients.

### Bioinformatics and Statistical Analysis

Both α-diversity (Simpson-reciprocal and Shannon indices) and β-diversity (Bray–Curtis distance) were examined using QIIME (Version 1.7.0). PCoA was used to reduce the dimension of the original variables with the Vegan and ggplot2 packages in R, while Analysis of similarity (ANOSIM) of the distance matrices in the vegan package in R was used to quantize the similarity and test the statistical significance between groups (Buttigieg and Ramette, 2014). Hierarchical clustering on the basis of similarities in the combination of variables was carried out using Pvclust in R. The microbiota were characterized using the linear discriminant analysis effect size (LEfSe) method for representative taxa discovery, emphasizing both significance and biological relevance (Segata et al., 2011). Functional composition of the gut metagenomes were predicted and profiled in accordance with the 16S rRNA gene sequences using PICRUSt with level III KEGG database pathways (Langille et al., 2013). Both PICRUSt and LEfSe were accomplished online^3^. A heatmap was created to express the results with the heatmap package in R. The microbiota features were further analyzed as categorical variables using an univariate logistic regression to screen risk factors. The optimal cut-off for each bacterial group was determined by ROC analysis. Variables with a *P* value < 0.1 on the univariate analysis were selected for further forward stepwise multivariate logistic regression to identify independent predictors. Odds ratios (ORs) were calculated with a 95% confidence interval (CI). The random forest (RF) algorithm was used to create the classification models. The optimal number of variables was determined by maximizing the area under the curve of the receiver operator characteristic (AUC) with the AUCRF package, then caret (v6.0.76) and random forest R package were used to build model. To avoid over-fitting of the data in the model, 10-time and 10-fold cross-validations were made. The resulting model was subsequently used for validation cohort.

All categorical data were presented as number of cases and percentages, while continuous data were shown as median with range. Categorical variables were compared by the Pearson’s chi-square (χ^2^) test, and continuous variables by Mann–Whitney *U* test where appropriate. Statistical analysis of the data was performed using SPSS (SPSS version 19, La Jolla, CA, United States). Wilcoxon rank sum test and Multiple hypothesis tests were used for analysis of continuous and categorical data and adjusted using the Benjamini and Hochberg FDR. The results with an FDR threshold lower than 0.1 were considered significant differences. Spearman’s rank test was used for correlation analysis, and a *P* value less than 0.05 was considered statistically significant.

## Results

### Characteristics of the Study Patients

Forty-one patients were included for discovery cohort, of which 22 patients developed metachronous adenoma (MA group), and the remaining 19 patients did not have any signs of metachronous adenoma [non-metachronous adenoma (nMA) group]. Demographic and clinical features between the two groups were summarized in Table 1. Body mass index (BMI) in the MA group was significantly greater than that of the nMA group (25.25 vs. 23.0; *P* < 0.05). Notably, the incidence of synchronous adenoma was significantly higher in the MA versus nMA groups (15/22 vs. 7/19; *P* < 0.05). No other significant differences between the two groups were observed. Information for every participant were supplied in Supplementary Table S1. Another 45 patients were included for validation cohort, 21 of which developed MA (Supplementary Table S2).

**TABLE 1:** Clinico-pathological characteristics of patients.

	MA (*n* = 22)	nMA (*n* = 19)	*P* value
**Gender**			
Female	12	6	0.139
Male	10	13	
**Age (years)^a^**	63 (58.5–68.75)	61.3 (53–68.5)	0.619
**BMI^a^**	25.25 (22.75–27.98)	23.0 (21.74–23.7)	0.011*
**Synchronous adenoma**			
Yes	15	7	0.045*
No	7	12	
**Bowel obstruction^d^**			
Yes	2	2	0.877
No	20	17	
**Hematochezia**			
Yes	11	11	0.613
No	11	8	
**Tumor size^ac^**	4 (3.6–4.2)	4 (3.1–4.75)	0.854
**Tumor location^b^**			
Left hemi-colon	7	2	0.171
Right hemi-colon	3	6	
Rectum	12	11	
**CEA^a^**	6.725 (2.38–14.30)	3.97 (2.37–12.83)	0.896
**CA 19-9^a^**	12.31 (7.15–65.44)	12.55 (10.99–20.06)	0.744
**Adjuvant therapy**			
Yes	13	12	0.790
No	9	7	
**TNM-stage**			
I	2	2	0.537
IIA	17	11	
IIIA	0	1	
IIIB	3	5	

*^*a*^Data shown as median (1st and 3rd quartile). ^*b*^Tumor location: splenic flexure, descending, sigmoid, rectosigmoid were classified as left hemi-colon; ileocecal, ascending, hepatic flexure, transverse were classified as right hemi-colon. ^*c*^Tumor size definition: maximum diameter. ^*d*^Bowel obstruction was defined when coloscopy cannot pass through the tumor obstruction.*

### Mucosal Microbial Diversity Is Significantly Associated With Metachronous Adenoma

**FIGURE 1:**
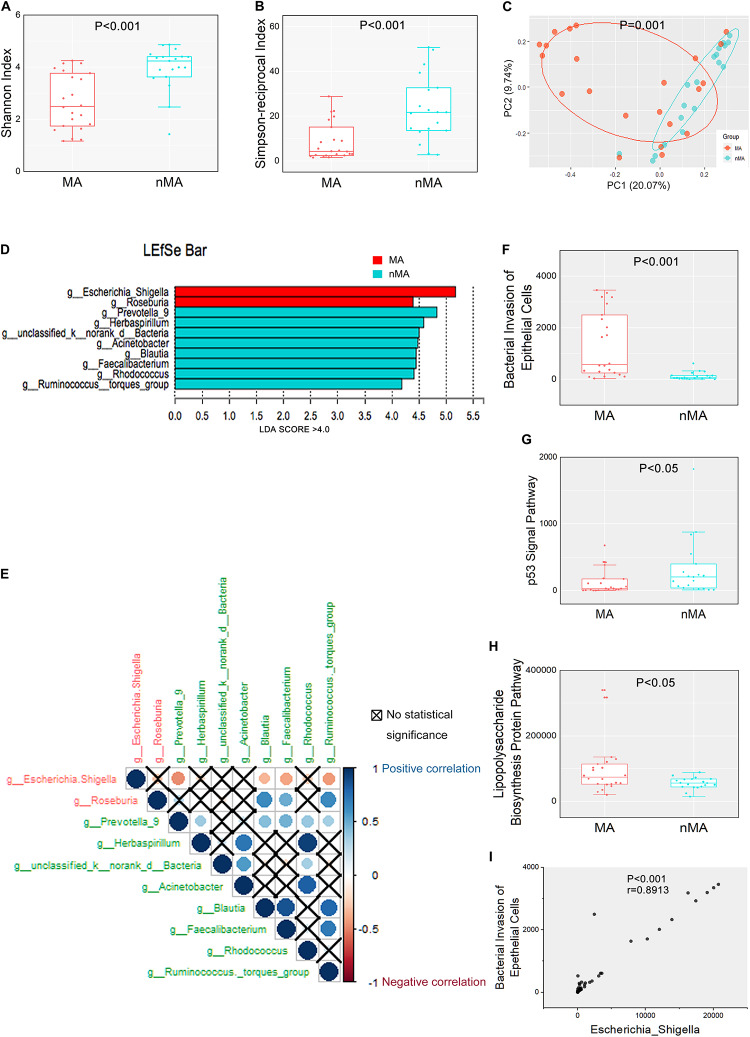
Mucosal microbiome diversity and communities are significantly different between MA and nMA. **(A,B)** α-diversity boxplot (Shannon and Simpson-reciprocal indices) of mucosal samples in MA and nMA groups. Boxes represented the 25th to 75th percentile of the distribution; the median was shown as a thick line in the middle of the box; whiskers extend to values with 1.5-times the difference between the 25th and 75th percentiles. **(C)** PCoA using Bray–Curtis of β-diversity in MA and nMA groups. **(D)** LDA score computed from features differentially abundant between MA and nMA in mucosal samples. The criteria for feature selection was log LDA score > 4. **(E)** Spearman correlations among two MA-enriched (red) and eight-nMA enriched (green) genera taxa in mucosal samples of CRC patients. Red dots indicated negative correlation, blue dots indicated positive correlation, cross indicated no significance (*P* > 0.05). **(F–H)** Boxplot of *bacterial invasion of epithelial cells pathway*, *Lipopolysaccharide biosynthesis protein pathway*, and *p53 signal pathway* between MA and nMA. *P* values were adjusted using the FDR correction. **(I)** Spearman correlation between *bacterial invasion of epithelial cells pathway* and relative abundance of *Escherichia–Shigella*.

We initially examined the correlation between mucosal microbial diversity and the development of MA. As shown in Supplementary Figure S1, the 16S rRNA gene-sequencing reads and depths were adequate. An analysis of the mucosal microbial diversity with two methodologies (Shannon and Simpson-reciprocal indices) showed that alpha-diversity of the mucosal microbiome was significantly higher in the nMA group compared with the MA group (*P* < 0.001 for each index) (Figures 1A,B). A principal coordinate analysis (PCoA) on genus level with Bray–Curtis metric distance was performed for comparison of β-diversity between the two groups. As shown in Figure 1C, a clear clustering between the MA and nMA groups was revealed, suggesting that the mucosa microbial communities exhibited phylogenetic closeness within each group (*P* = 0.001). Importantly, we excluded the possibility of any other potential contributors to the microbial diversity, such as clinical-pathological features, synchronous adenoma, BMI, sex, and adjuvant therapies (Supplementary Figure S2).

### Mucosal Microbial Composition and Function in the MA Group Differs Significantly From Those in the nMA Group

We next determined if there were differences in the mucosal microbial composition between the MA and nMA patients using linear discriminant analysis of effect size (LEfSe). After bacterial taxa with relative abundance <0.5% were excluded for comparison, 10 taxa showed differentiated distribution with LDA score > 4.0 on genus level. The MA group exhibited a predominance of *Escherichia–Shigella* and *Roseburia*, while the nMA group had a predominance of *Prevotella_9*, *Herbaspirillum*, *unclassified_k_norank_d_Bacteria*, *Acinetobacter*, *Blautia*, *Faecalibacterium*, *Rhodococcus*, and *Ruminococcus_torques_group* (Figure 1D). We then examined the potential interactions among these 10 taxa with Spearman rank test. As a result, *Escherichia–Shigella* was always negatively correlated (red dots) with others taxa, while the genera enriched in the nMA group (green text) positively correlated (blue dots) with each other (Figure 1E).

Further analysis showed there were four taxa on the phylum level and six taxa on the family level that predominated in the two groups with LDA score > 4.0 (Supplementary Figure S3). We then interrogated whether the mucosal microbiome can be segregated using BMI or synchronous adenoma as grouping variables. Only one and two predominate genera with LDA score > 4.0 were found, respectively, based on BMI (high or normal) and synchronous adenoma status (Supplementary Figure S4), indicating that MA rather than BMI or synchronous adenoma was the main explanation to the different microbiota composition between the two groups.

The functions of the gut microbiota were predicted using the PICRUSt analysis. 16S rRNA gene sequencing data were categorized into 328 KEGG functional pathways; pathways present in <10% of participants were removed, leaving 284 KEGG pathways for comparation. Fifty five pathways were differentially enriched between the two groups (*P_*fdr*_* < 0.1) (Supplementary Figure S5). We observed significant upregulation of *bacterial invasion of epithelial cells pathway* and *lipopolysaccharide biosynthesis protein pathway* in the MA group compared with the nMA group (*P*_*fdr*_ < 0.1). On the contrary, *p53 signal pathway* was downregulated in the MA group (*P*_*fdr*_ < 0.1) (Figures 1F–H). Specifically, the potential pathogenic bacteria *Escherichia–Shigella* was positively correlated with *bacterial invasion of epithelial cells pathway* (*r* = 0.89, *P* < 0.01) (Figure 1I).

### Microbiota Profiles of the Mucosal and Fecal Samples

**FIGURE 2:**
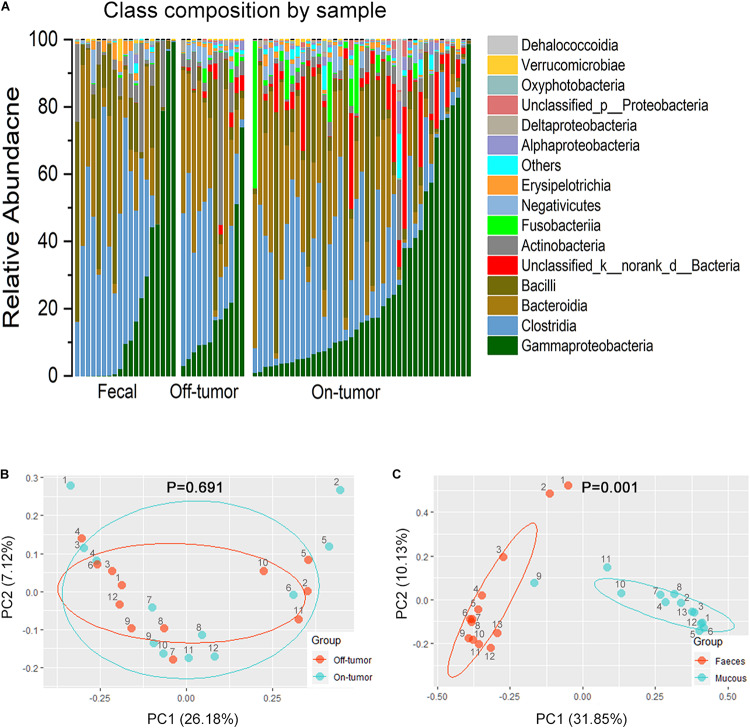
Fecal and off-tumor samples. **(A)** Bar plots of the class taxonomic levels of microbiota in fecal, off-tumor and on-tumor samples. Relative abundance is plotted for each samples. **(B)** PCoA using Bray–Curtis of β-diversity between on- and off-tumor mucosal samples. **(C)** PCoA using Bray–Curtis of β-diversity between fecal and mucosal samples.

Bar plots of the class taxonomic levels showed Gammaproteobacteria and Clostridia as the top two classes with higher relative abundance in all samples. *P < 0.05, different from controls by Wilcoxon rank-sum test or Chi-squared test for continuous or categorical variables, respectively. The microbiota composition was similar between on-tumor and off-tumor mucosal samples, whereas fecal samples showed independent features without detecting of *unclassified_k__norank_d__Bacteria* and *Fusobacteriia* (Figure 2A). Despite the collective differences between subjects with MA and nMA, the microbiota associated with on-tumor and off-tumor tissues in the same individual (*n* = 12) did not differ significantly in PCoA (Figure 2B) (*P* = 0.691). Hierarchical-Clustering analysis with Bray–Curtis distance indicated no apparent difference between the paired On/Off mucosal samples in the same individual (Supplementary Figure S6). On the contrary, fecal and mucosal samples in the same individual showed obviously different in PCoA (Figure 2C) (*P* = 0.001), paired fecal and mucosa samples within the same individual did not close to each other (Supplementary Figure S7).

Next, we assessed whether fecal microbiota profiles could reflect the difference between MA (*n* = 11) and nMA (*n* = 8). As expected, fecal microbiota profiles in the MA and nMA patients differed significantly in PCoA analysis (Supplementary Table S3 and Supplementary Figure S8) (*P* = 0.003). The microbiota of the fecal samples in LEfSe analysis by MA status produced five genera with LDA score > 4.0, with *Escherichia–Shigella*, *Blautia*, and *Ruminococcus_torques_group* profiles consistent with the findings of the mucosal profiling (Supplementary Figure S9). These results indicated that even though fecal microbiota do not corresponded to mucosa microbiota and only partially reflect the microbiota at the mucus layer, differences due to disease status are still evident.

### Gut Microbiota Variation of MA May Still Exist to Some Degree in Patients After Surgery

Our previous cross-sectional study showed significant difference in post-operative fecal microbiota between patients with and without MA, and the alterations in the gut microbiota was associated with the disease progression in health-adenoma-carcinoma sequence (Feng et al., 2015), indicating that patients with occurrence of metachronous had more “carcinoma-like” gut microbiota compared to clear-intestine patients. Intrigued by these pervious findings, we examined if there was an association between pre- and post-operative patients fecal microbiota on MA profile. To this end, we applied conjoint analysis by importing our previous 16S rRNA gene sequence data of fecal samples, assigned as post-operative cohort. The samples from this study were assigned as pre-operative cohort accordingly.

**FIGURE 3:**
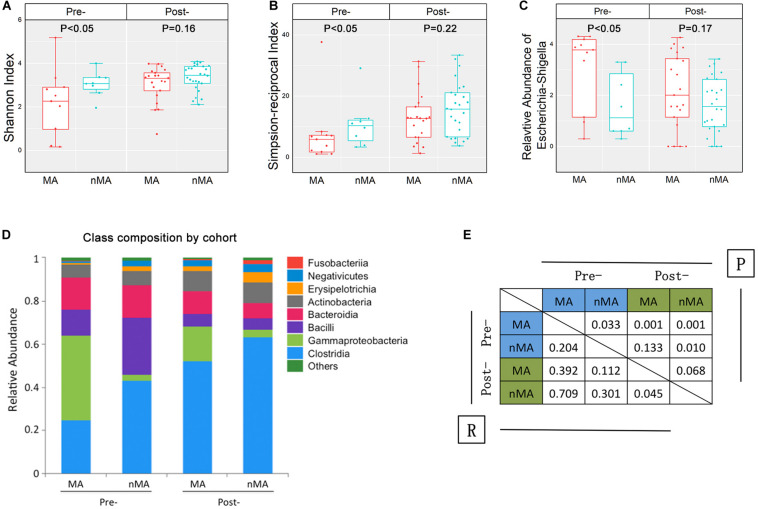
Fecal microbiota in CRC patients and CRC patients after surgical therapy. **(A,B)** α-diversity boxplot (Shannon and Simpson-reciprocal indices) of fecal samples. **(C)** Boxplots of relative abundance of fecal *Escherichia–Shigella*; boxplot illustration was provided in Figure 1. **(D)** Bar plots of the class taxonomic levels of fecal microbiota. Relative abundance is plotted for each group. **(E)** ANOSIM result between fecal samples of groups. *R* value indicated the strength of the factors on the samples, while give *P*-value indicated the significance levels.

The overall α-diversity of post-operative patients (*n* = 47) was higher than that of pre-operative patients (*n* = 19) (data not shown). Similarly, α-diversity of the fecal samples were higher in the nMA patients (*P* < 0.05 for both Shannon and Simpson-reciprocal indices). For post-operative patients, α-diversity was higher in the nMA patients, whereas the difference was not statistically significant (*P* > 0.05 for both Shannon and Simpson-reciprocal indices) (Figures 3A,B). Next, *Escherichia–Shigella* was selected, as it was highly enriched and relatively abundant in both the mucosal and fecal samples in the MA patients (*P* < 0.05). In addition, this difference was also found in post-operative patients without reaching statistical significance (Figure 3C). Bar plots of the class taxonomic levels showed a difference in the microbiota composition between the MA and nMA patients, as well as between the post-MA and post-nMA patients. It was worth noticing that the microbiota composition of the MA patients was similar to that of the post-MA patients, while that of nMA was more similar to post-nMA (Figure 3D).

ANOSIM was performed to determine the β-diversity between groups, in which ANOSIM gave a *P* value (i.e., significance levels) and a *R* value (i.e., the strength of the factors on the samples). As a result, the *R* value between the MA and nMA groups was 0.204 (*P* = 0.033), while *R* value between the post-MA and post-nMA groups was 0.045 (*P* = 0.068), indicating that the discrepancy between patients with and without MA was less obvious in patients undergone surgery compared to untreated patients. *R* values between post-nMA and MA or nMA (*R* = 0.709 or *R* = 0.301; *P* = 0.001 or *P* = 0.01) were higher than those between post-MA and MA or nMA (*R* = 0.392 or *R* = 0.112; *P* = 0.001 or *P* = 0.133) (Figure 3E), suggesting that gut microbiota of post-operative patients without MA to be more different from CRC patients, especially from CRC patients who develop MA. Collectively, these results indicated that gut microbiota-based discrepancy between patients with and without MA remained in post-operative patients.

### Pre-operative Gut Microbiota-Based Random Forest Algorithms and Scoring System in the Prediction of Metachronous Adenoma in CRC Patients After Surgery

Firstly, 7 of 10 predominance bacterial genera in MA and nMA identified by LEfSe analysis, together with BMI, and synchronous adenoma were applied to logistic regression. *Herbaspirillum*, *Rhodococcus*, and *Prevotella_9* were excluded, as they were not detectable in more than five patients. All these variables were identified as significant risk factors for MA by univariate logistic regression (*P* ≤ 0.1) (Table 2), then multivariate logistic regression analysis was applied for independent risk factor validation. As shown in Table 3, the predominant bacterial genera, including *Escherichia–Shigella* and *Acinetobacter*, as well as BMI were identified as independent risk factors for MA (*P* < 0.05), with a good ability for differentiating MA from nMA (AUC, 0.935).

**TABLE 2:** Univariate logistic regression predicting MA.

	Cut-off value	OR	95% CI	*P* value
*Escherichia–Shigella*	564.5	10.000	2.350-42.547	0.002*
*unclassified_k_norank_d_Bacteria*	147	0.206	0.037-1.131	0.069
*Faecalibacterium*	608.5	0.172	0.044-0.672	0.011*
*Ruminococcus_torques_group*	10.5	0.097	0.011-0.871	0.037*
*Blautia*	732.5	0.065	0.007-0.593	0.015*
*Acinetobacter*	28	0.056	0.006-0.492	0.009*
*Roseburia*	55	0.172	0.044-0.672	0.011*
Synchronous adenoma		3.673	1.007-13.395	0.049*
BMI		1.396	1.069-1.824	0.014*

**TABLE 3:** Multivariable logistic regression model predicting MA.

	OR	95% CI	*P* value
*Escherichia–Shigella*	53.254	3.338-849.676	0.005*
*Acinetobacter*	0.026	0.001-0.477	0.014*
BMI	1.684	0.993-2.855	0.053

**FIGURE 4:**
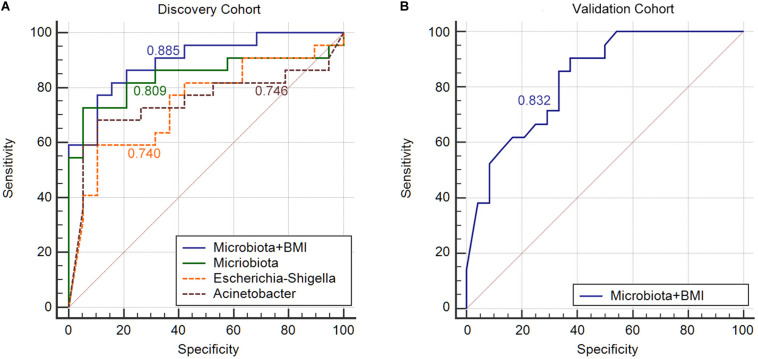
Gut microbiota signature can be used to discriminate between MA patients from nMA patients. **(A)** ROC analysis in discovery cohort with *Escherichia–Shigella* along, *Acinetobacter* along, combination of two genera (Microbiota), and bacterial genera together with BMI (Microbiota + BMI). **(B)** ROC analysis with bacterial genera together with BMI (Microbiota + BMI) in validation cohort.

Next, we constructed an RF algorithm using the relative abundance of the gut microbial populations with or without the clinical risk factors to predict MA. To determine the potential of bacterial taxa in discriminating MA, we aimed to identified a minimal set of bacterial genera that maximally differentiated nMA from MA. Firstly, 10 predominant bacterial genera produced by LEfSe were initially screened, and a combination of *Escherichia–Shigella* and *Acinetobacter* optimized the performance of RF model (Supplementary Figure S10), and thus were used to generate a new model. 10-times and 10-fold cross-validations were conducted to optimize the model in case of over-fitting. As shown in Figure 4, the AUC for the model was 0.809 and higher than *Escherichia–Shigella* or *Acinetobacter* alone in predicting MA (Figure 4A). Considering the potential value of some clinical factors in the prediction of MA, we hypothesized that the predominant bacterial populations and clinical factors in combination could generate a more precise RF model. To test the hypothesis, the independent clinical risk factors, including synchronous adenoma and BMI (Supplementary Figure S11), together with the predominant bacterial populations, *Escherichia–Shigella* and *Acinetobacter*, were used to build a new RF model. The AUC for the RF model was 0.885, which was greater than the AUC for the RF model using predominant bacterial populations alone (Figure 4A). This result indicated that, in addition to gut microbiota, clinical features of patients possessed additional predictive ability on MA. The RF model were further tested on fecal and off-tumor samples, the AUC was 0.835 and 0.889, respectively (Supplementary Figures S12, S13), suggesting that fecal and off-tumor mucosal samples can be used for MA prediction as well. However, the AUC for the RF model was 0.61 on post-operative fecal samples (Supplementary Figure S14). Finally, the RF model was applied for discovery cohort and got a AUC of 0.832 (Figure 4B).

In order to further validated the specificity of our RF model, we applied the RF model to predict local recurrence of colon cancer with previous published data (Bullman et al., 2017). The AUC value was 0.546, which indicated a poor predict ability for local recurrence (Supplementary Figure S15).

Finally, we developed a risk score for MA, which utilized the two predominant bacterial populations and the two clinical features. *Escherichia–Shigella*, BMI and synchronous-adenoma were risk factors, and the presence of each one was assigned one point, while the absence of beneficial factor, *Acinetobacter*, was scored one point. The cut-off values were determined by ROC analysis in the discovery cohort and applied the same value for the validation cohort to avoid over-fitting. As a result, the total risk scores ranged from zero to four points, and the risk score showed an AUC of 0.94 and 0.835 for the prediction of MA in discovery and validation cohort. Further, the presence of two or more risk factors in discovery cohort had a sensitivity and specificity of 90.9% and 89.5%, but specificity in validation cohort was 33.3% (Table 4).

**TABLE 4:** Sensitivity, specificity, PPV and NPV of the risk score based on predominant presence of the risk factors.

Risk score	Discovery cohort					Validation cohort				
	Sensitivity (%)	Specificity (%)	PPV	NPV	MA rate*	Sensitivity (%)	Specificity (%)	PPV	NPV	MA rate*
0	100	0	53.7	/	0 (0/6)	100	0	46.67	/	0 (0/2)
1	100	31.6	62.9	100	15.38 (2/13)	100	8.33	48.84	100	14.28 (1/7)
2	90.9	89.5	90.9	89.5	83.3 (10/12)	95.24	33.33	55.56	88.89	23.08 (3/13)
3	45.5	100	100	61.3	100 (5/5)	80.95	75	73.91	81.82	66.67 (10/15)
4	22.7	100	100	52.8	100 (5/5)	33.33	95.83	87.5	62.16	87.5 (7/8)

## Discussion

We conducted the first study, to the best of our knowledge, to assess the correlation between pre-operative gut microbiota and MA among Chinese CRC patients after surgery and to develop novel microbiota-based predictive models. The novel findings are summarized as follows: (1) There was a significant correlation between pre-operative gut microbiota and the development of MA among CRC patients after surgery. (2) Specific members of the predominant gut microbiota, including *Escherichia–Shigella* and *Acinetobacter*, were identified as independent risk factors for MA. (3) The microbiota-based RF model was established utilizing these specific members of predominant gut microbiota combined with independent clinical risk factors (BMI) and the status of synchronous adenoma, showing a good performance (AUC, 0.885) to predict MA among CRC patients after surgery. (4) The microbiota-based RF model exhibited good ability in the prediction of MA using fecal and off-tumor samples (AUC, 0.835 and 0.889, respectively). (5) A risk-scoring system was proposed with four independent predictive factors got an AUC of 0.94 and 0.835 for the prediction of MA in discovery and validation cohort.

Colonoscopic mucosa biopsies were used rather than an intra-operative specimen, because we thought the microbiota of samples from resected tumor after operation may be disturbed by clinical intervention, such as the preventive antibiotics application before operation. A clear clustering between the MA and nMA patients was observed. α-diversity of the mucosal and fecal samples were both lower in the MA group. As low diversity microbiota indicated unstable ecosystem, one piece of evidence that has emerged from many large surveys of gut microbial communities is that low microbial diversity is almost invariably associated with disease (Round and Palm, 2018).

It was noticed that there were predominated bacterial taxa in both MA and nMA, respectively. Specifically, we found the genera enriched in nMA group positively correlated each other. This co-abundance groups (CAG) of bacterial taxa resembled the previously formulated concept of enterotypes. The bacterial taxa belonged to one CAG may relate to each other not only quantitatively but also functionally (Flemer et al., 2017). *Escherichia–Shigella* was identified as the most abundant genus in the MA patients. *Escherichia* comprises eight species, including the well-known *Escherichia coli* (*E. coli*). Although *Shigella* is technically a independent genus with four species, they are inseparable from *E. coli* in terms of 16S rRNA gene DNA sequence, so they are commonly bracketed together and named *Escherichia–Shigella* in 16S rRNA gene-based microbiota studies. All these species belong to the Enterobacteriaceae, which was highly enriched in the MA patients as well. *Escherichia–Shigella* has been shown to produce Colibactin, which is encoded by polyketide synthase (*pks*) genotoxicity island (Nougayrède et al., 2006). Colibactin possesses the capacity to damage DNA and lead to CRC development (Wu et al., 2009; Arthur et al., 2012). Mucosa-associated *E. coli* has been found to be significantly more prevalent in CRC tissue and correlates with tumor stage and prognosis (Bonnet et al., 2014).

*E. coli* and *Shigella* have been shown to increase intestinal permeability in this intestinal disorder, likely due to down-regulation of tight junction proteins (Cinova et al., 2011). Our study demonstrated that *Escherichia–Shigella* was positively correlated with *bacterial invasion of epithelial cells pathway*, which was also enriched in the MA patients as identified by PICRUSt method. The *bacterial invasion of epithelial cells pathway* indicates that the potential pathogens such as *Escherichia–Shigella* and *Enterococcus* could adhere the surface of host cells, cross host epithelial barriers, and get access to internal tissues, thereby promoting their dissemination inside the host (Ribet and Cossart, 2015).

It was striking that there was high similarity in the mucosal microbiota of paired on–off tumor samples with regard to overall composition of the microbiota. In contrast, paired fecal and mucosal samples had lower similarity. These findings were consistent with a previous study (Nakatsu et al., 2015). We found that microbiota in the fecal samples can be also separated between the MA and nMA groups. As such, even though fecal microbiota differed from and may only partially reflects the microbiota at the mucus layer, differences due to MA status are still evident. Unlike mucosal samples, which mainly reflected the local microbiota, the fecal samples may be a representative for the whole gut environment. It is possible that except for the lesion site, other sites of the colon may also possess more CRC-related bacteria in the MA patients, compared to the nMA patients.

Our previous cross-sectional study showed differences in post-operative fecal microbiota between patients with and without MA (Jin et al., 2019). We wonder whether such difference could exist in the pre-operative fecal samples. As observed in our study, similar to pre-operative CRC cohort, lower microbiota diversity, and higher abundance CRC-related bacterial taxa were characteristics for MA in the post-operative cohort, but not obvious as pre-operative cohort. ANOSIM results also showed the distance value between MA and nMA was high in pre-operative cohort. Collectively, these findings suggest residual microbiota features for MA still exist in post-operative cohort.

In this study, we identified novel microbiome biomarkers for prediction of the MA. It is important to highlight that MA is a complex disease that occurs as a combination of microbial colonization, patient genetic background, and other environment factors. Given that, we established the RF model utilizing the gut microbiota together with the clinical risk factors to predict MA. We observed that the key predictor was *Escherichia–Shigella* in this model which was in agreement with logistic regression result, showing that *Escherichia–Shigella* was an independent risk factors with an overt OR value of 53.254. Although synchronous adenoma was not included in the RF model, in view of it as a risk factor for MA and in order to translate our result to clinical application, we developed a risk score based on presence of the negative prognostic genus *Escherichia–Shigella*, absence of the positive prognostic genus *Acinetobacter*, together with high BMI and the traditionally accepted risk factors, synchronous adenoma. The specificity was lower in the validation cohort; one explanation maybe the discovery cohort derived cut-off value was not optimized enough, but there was still a high sensitivity in validation cohort and the overall AUC value was reasonable. As expected, the RF model performed well for off-tumor mucosal and fecal samples. The RF model cannot predict local recurrence with data imported from Bullman et al. (2017) study, which may indicate the specificity of our predict model. Although this clinical condition is an excellent model for investigating whether dysbiosis precedes MA, we can’t draw conclusions regarding the causality on the basis of our data. We wonder if CRC patients at high risk for MA could be identified pre-operatively by gut microbiota; an individual post-operative surveillance plan can be made to prevent the occurrence of metachronous CRC.

Our study may have a number of limitations. Firstly, patients were followed up, but mucosal or fecal samples were not collected after surgery, for which we cannot make a before–after analysis in the same cohort of patients. But, we made conjoint analysis with previous data of another cohort patients. Secondly, the sample size was relatively small, and the predicted potential of the selected biomarkers should be evaluated in an independent cohort. Although no external cross-validation was achieved in this study, sufficient internal cross-validation with different samples was made. Thirdly, the patients were followed up with for 12 months, so we could only observe MA development, but not metachronous carcinoma.

The findings have demonstrated that specific members of the dominant gut microbiota as non-invasive biomarkers for prediction of MA or CRC after surgical resection. The newly established RF algorithm and the risk-scoring system have a good ability to predict the development of MA after surgical resection, and therefore, the novel approaches hold potential to guide individual post-operative surveillance plan for CRC patients in future clinical application.

## Data Availability Statement

The raw sequences have been deposited in the NCBI Sequence Read Archive (Nos. PRJNA594545 and PRJNA573487), and the necessary metadata can be found at https://www.ncbi.nlm.nih.gov/Traces/study/ by searching the respective SRA study accession.

## Ethics Statement

The study protocol was reviewed and approved by the Research Ethics Committee of the First Affiliated Hospital of Harbin Medical University. Each patient had provided a written informed consent. The study involving human subjects was strictly performed according to international guidelines regarding the conduct of clinical trials. This study was registered at ClinicalTrials.gov (NCT03667495).

## Author Contributions

YL and YW conceived the study design. RG and LL recruited and followed up the patients. XJ coordinated with patients transported patient samples. YL and FZ performed the sequencing analysis. WY and SW contributed to the data analyses. XG and GG maintained patient records. YL and YW drafted the manuscript. All authors read and approved its final version.

## Conflict of Interest

The authors declare that the research was conducted in the absence of any commercial or financial relationships that could be construed as a potential conflict of interest.

## Footnotes

1 http://drive5.com/uparse/

2 http://rdp.cme.msu.edu/

3 http://huttenhower.sph.harvard.edu/galaxy
