# Supplementary material for: Gut Microbiota-Based Algorithms in the Prediction of Metachronous Adenoma in Colorectal Cancer Patients Following Surgery
Source: Front Microbiol. 2020 Jun 12;11:1106. doi: 10.3389/fmicb.2020.01106 (PMC7303296; doi:10.3389/fmicb.2020.01106)
Supplement: Supplementary file 1 [file Data_Sheet_1.PDF]

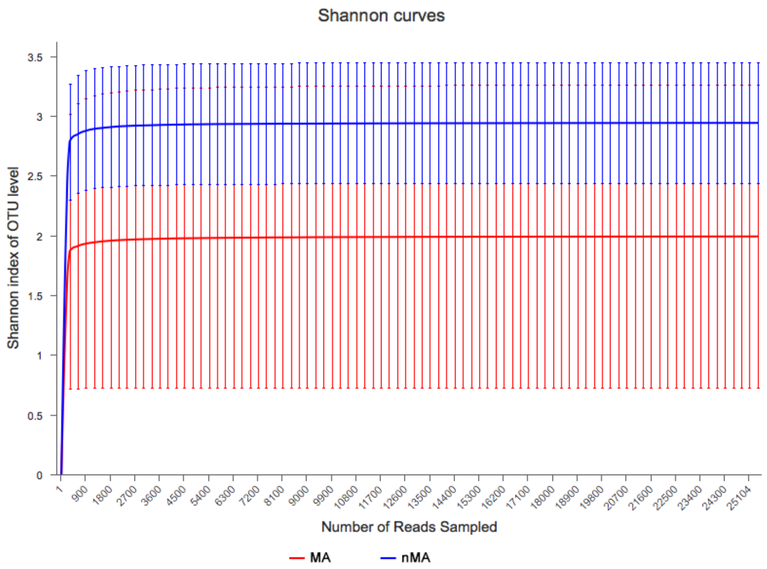

Fig. S1: Rarefaction curve reached plateau, indicated the sequencing depth was adequate.

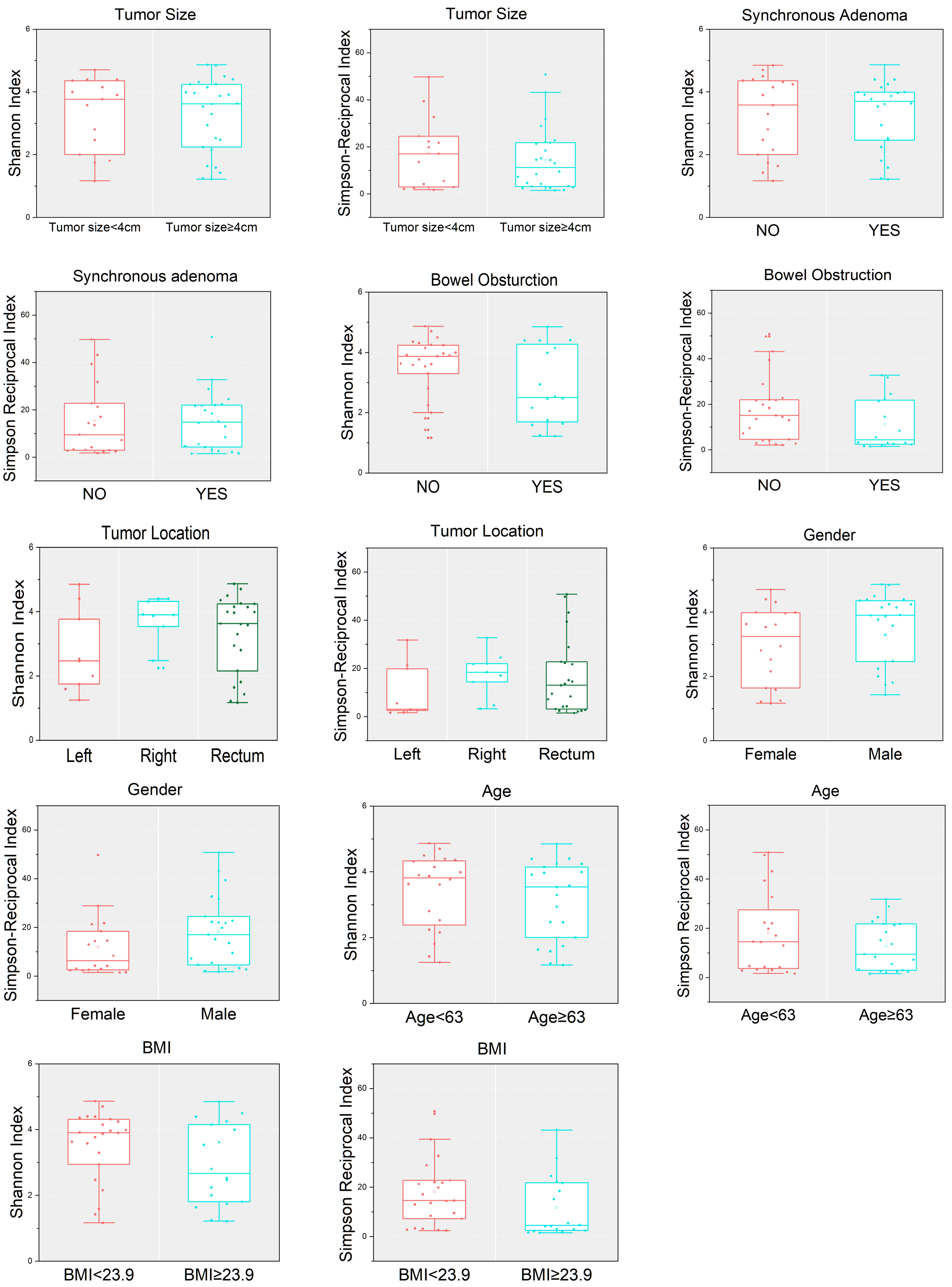

Fig. S2: Alpha diversity boxplot (Shannon and Simpson-reciprocal) of mucosal samples classified by clinical variables (tumor size, synchronous adenoma, bowel obstruction, tumor location, gender, age and BMI), no relation was found between alpha-diversity and clinical variables.

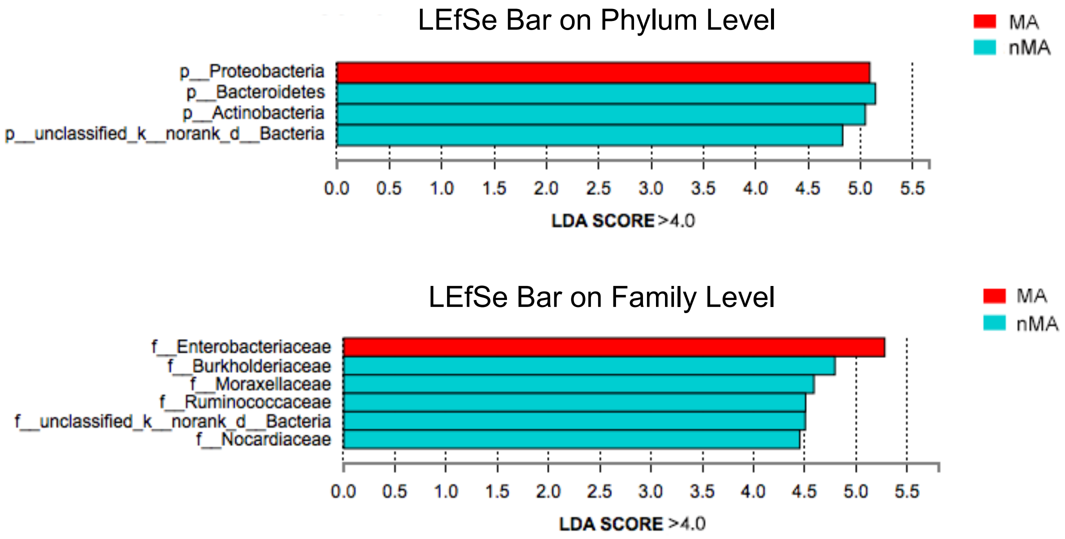

Fig. S3：LDA score computed from features differentially abundant on phylum and family level between MA and nMA in mucosal samples. The criteria for feature selection was LDA score>4.

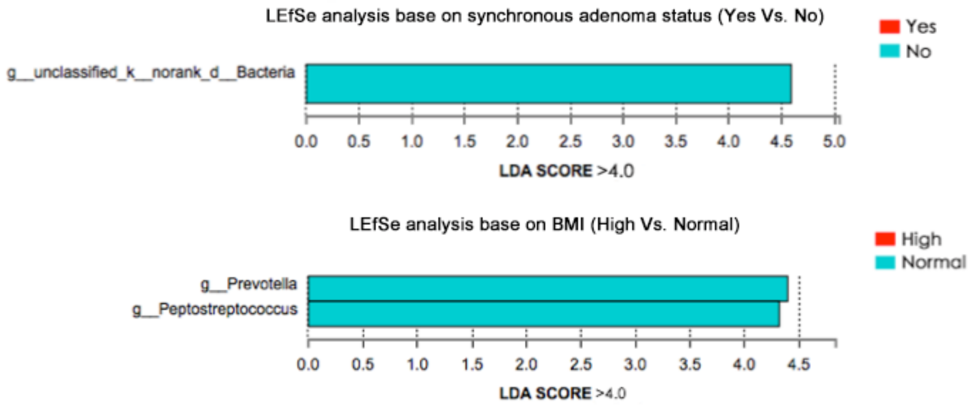

Fig. S4：LDA score computed from features differentially abundant between patients with and without synchronous adenoma, with high and normal BMI. The criteria for feature selection was LDA score>4.

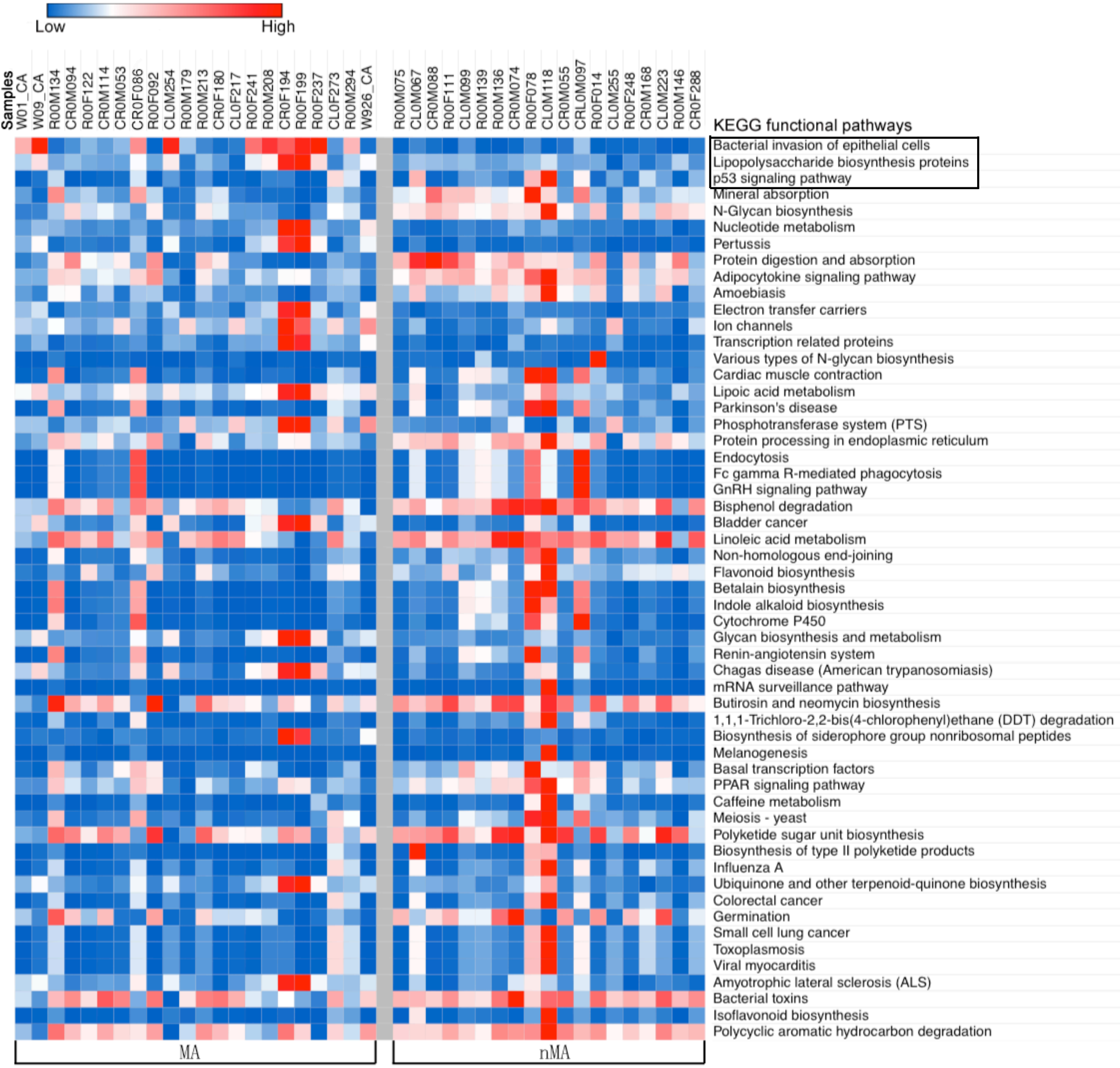

Fig. S5: Heatmap of selected most differentially (P_{fdr}<0.1) abundant features of the KEGG functional pathways. The red color represents high abundance and blue represents low abundance. Rows indicated KEGG pathways while columns indicated samples.

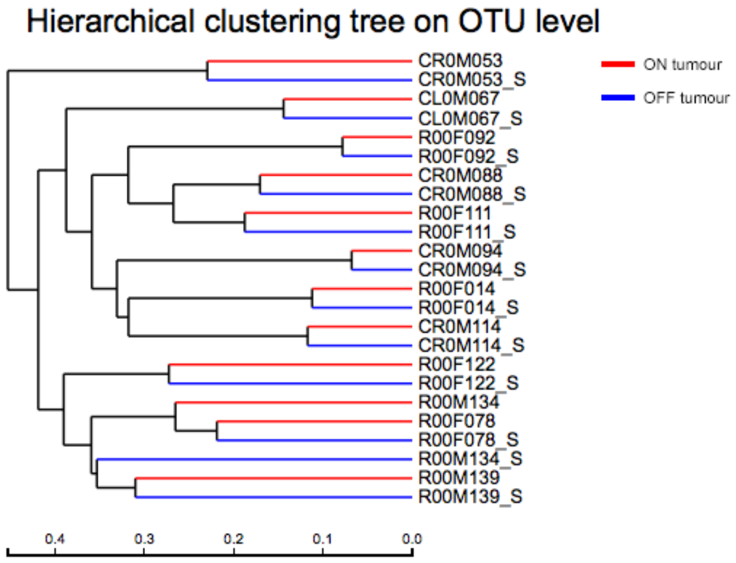

Fig. S6: Hierarchical clustering for 12 participants with paired ON tumor (red lines) and OFF tumor (blue lines) samples using Bary-Curtis distance.

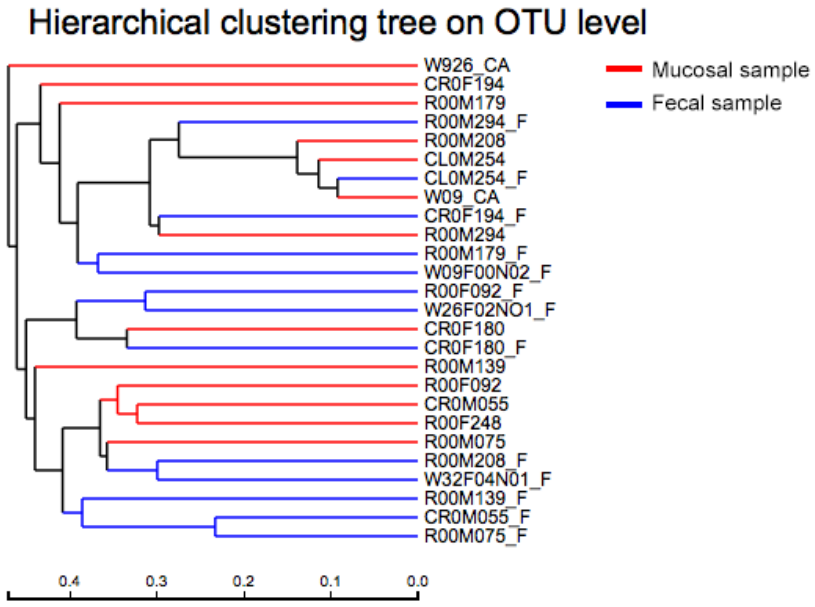

Fig. S7: Hierarchical clustering for 13 participants with paired mucosal (red lines) and fecal (blue lines) using Bary-Curtis distance.

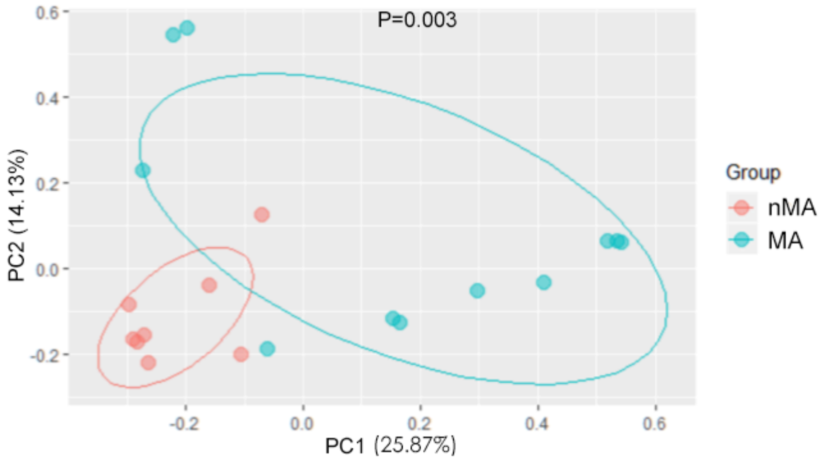

Fig. S8: PCoA using Bary-Curtis of beta diversity between MA and nMA fecal samples.

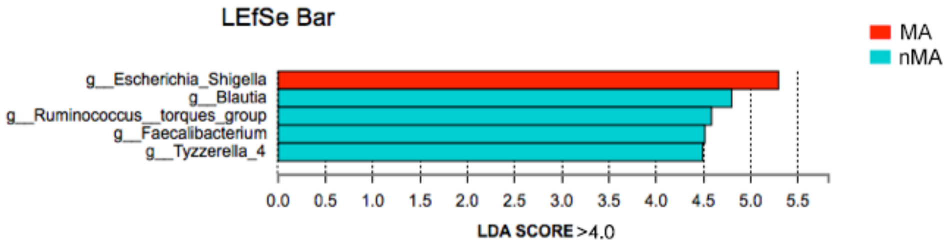

Fig. S9: LDA score computed from features differentially abundant on genus level between MA and nMA in fecal samples. The criteria for feature selection was log LDA score > 4.

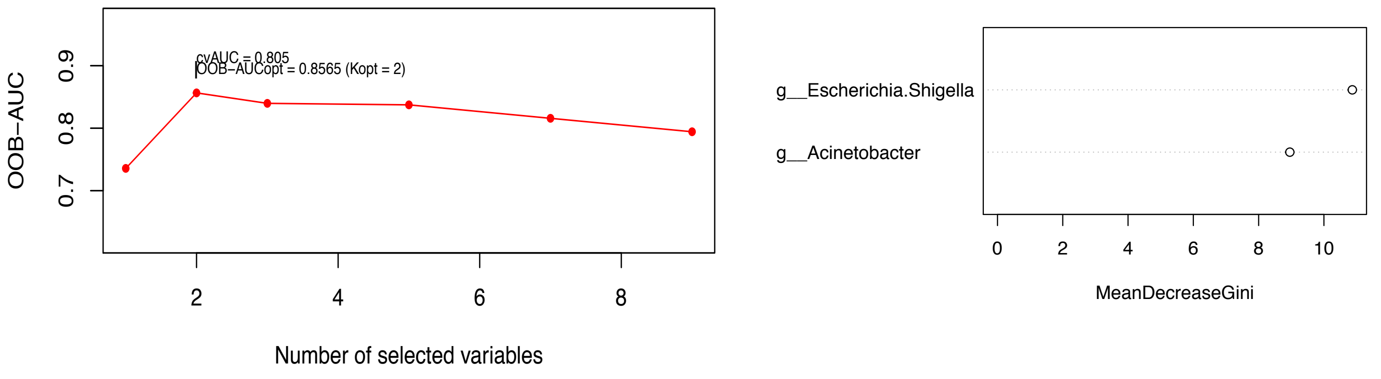

Fig. S10:The relationship between number of variables and AUC value in the RF model, the mean decreased Gini index of the top two variable in the RF model (Microbiota).

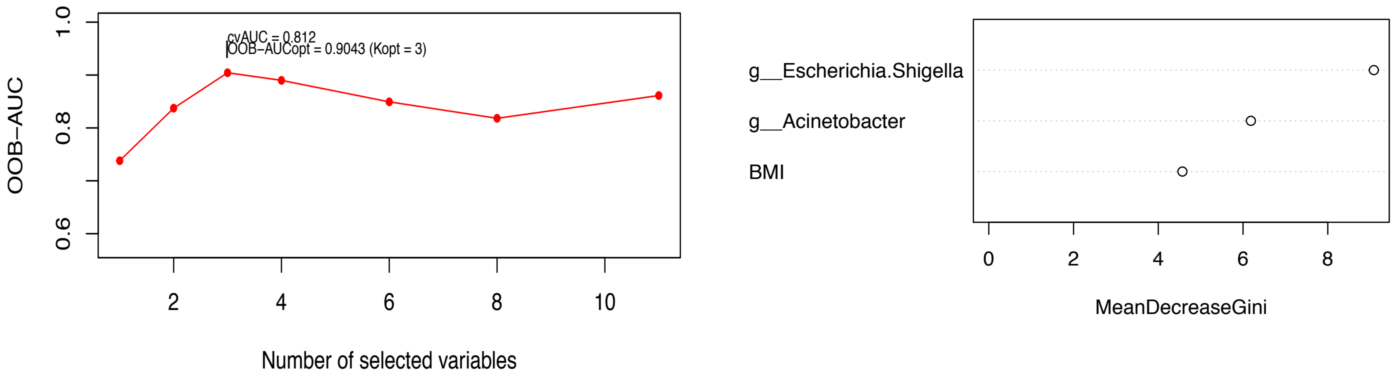

Fig. S11:The relationship between number of variables and AUC value in the RF model, the mean decreased Gini index of the first three variable in the RF model (Microbiota +Other risk factors).

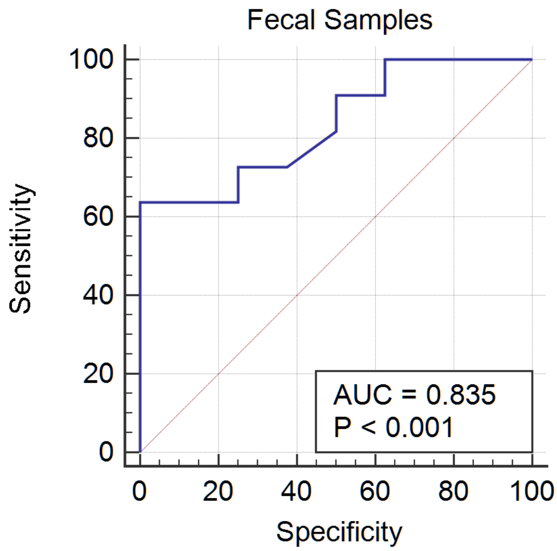

Fig. S12: ROC analysis of the RF model on fecal samples.

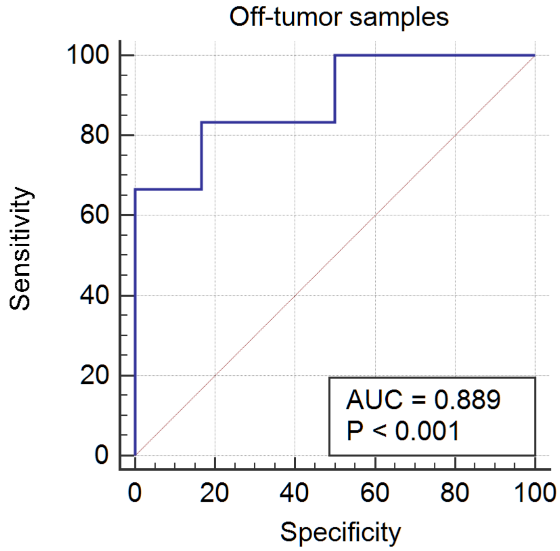

Fig. S13: ROC analysis of the RF model on Off-tumor samples.

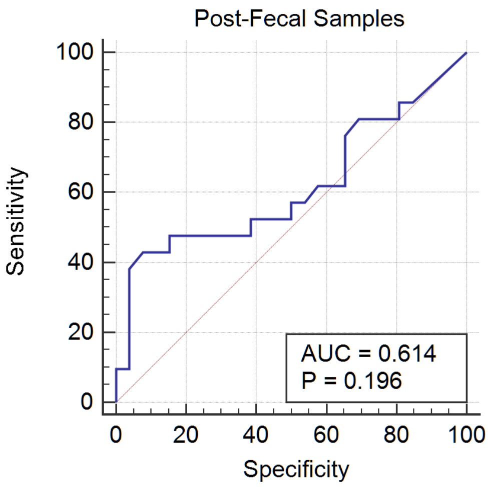

Fig. S14: ROC analysis of the RF model on fecal samples of patients undergone surgery.

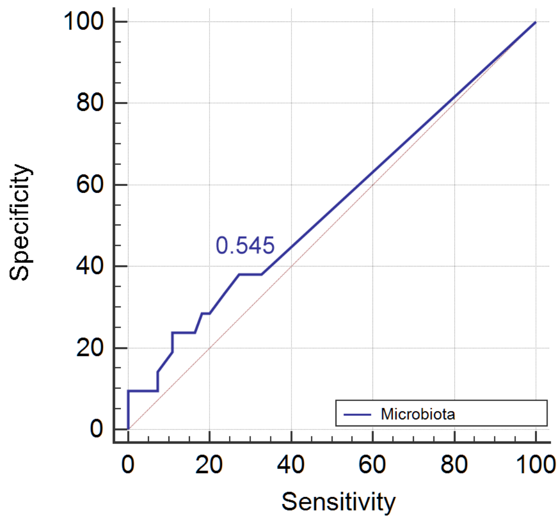

Fig. S15: ROC analysis of the RF model on local recurrence prediction with previous published data.
