# Supplementary material for: Gut Microbiota-Based Algorithms in the Prediction of Metachronous Adenoma in Colorectal Cancer Patients Following Surgery
Source: Front Microbiol. 2020 Jun 12;11:1106. doi: 10.3389/fmicb.2020.01106 (PMC7303296; doi:10.3389/fmicb.2020.01106)
Supplement: Supplementary file 2 [file Data_Sheet_2.docx]

**Correlation matrix**

mydata<- read.csv("genus_lefse.csv",header = T,row.names = 1)

str(mydata)

library(Hmisc)

res2 <- rcorr(as.matrix(mydata),type = "spearman")

res2

library(corrplot)

# Insignificant correlation are crossed

corrplot(res2$r, type="upper", order="original",

p.mat = res2$P, sig.level = 0.05,tl.pos = "td",tl.cex=0.55,

tl.col = c("#FF6666","#FF6666",

"#009933","#009933","#009933","#009933","#009933"

,"#009933","#009933","#009933"),cl.length = 5,cl.cex = 0.6,cl.ratio=0.2,cl.align.text="r", cl.offset = -0.03)

**PCoA**

otu <- read.delim('F-M.txt', row.names = 1, sep = '\t', stringsAsFactors = FALSE, check.names = FALSE)

otu <- data.frame(t(otu))

group <- read.delim('F-M_group.txt', sep = '\t', stringsAsFactors = FALSE)

library(vegan)

distance<- vegdist(otu,method='bray')

pcoa <- cmdscale(distance, k = (nrow(otu) - 1), eig = TRUE)

ordiplot(scores(pcoa)[ ,c(1, 2)], type = 't')

summary(pcoa)

pcoa$eig

point <- data.frame(pcoa$point)

pcoa_eig <- (pcoa$eig)[1:2] / sum(pcoa$eig)

sample_site <- data.frame({pcoa$point})[1:2]

sample_site$ID <- rownames(sample_site)

names(sample_site)[1:2] <- c('PCoA1', 'PCoA2')

sample_site <- merge(sample_site, group, by = 'ID', all.x = TRUE)

#sample_site$Group <- factor(sample_site$Group, levels = c("YES","NO"))

library(plyr)

group_border <- ddply(sample_site, 'Group', function(df) df[chull(df[[2]], df[[3]]), ])

library(ggplot2)

pcoa_plot <- ggplot(sample_site, aes(PCoA1, PCoA2, group = Group)) +

theme(panel.grid = element_line(color = 'gray', linetype = 2, size = 0.1), panel.background = element_rect(color = 'black', fill = 'transparent'), legend.key = element_rect(fill = 'transparent')) +

geom_vline(xintercept = 0, color = 'gray', size = 0.4) +

geom_hline(yintercept = 0, color = 'gray', size = 0.4) +

geom_polygon(data = group_border, aes(fill = Group)) +

geom_point(aes(color = Group, shape = Group), size = 2.5, alpha = 0.8) +

scale_shape_manual(values = c(16, 16)) +

scale_color_manual(values = c('#33CCCC', '#FF3300')) +

scale_fill_manual(values = alpha(c("#00FFFF", "#FF0066"), .2)) +

guides(fill = guide_legend(order = 1), shape = guide_legend(order = 2), color = guide_legend(order = 3)) +

labs(x = paste('PCoA axis1: ', round(100 * pcoa_eig[1], 2), '%'), y = paste('PCoA axis2: ', round(100 * pcoa_eig[2], 2), '%'))

pcoa_plot

**Hierarchical Clustering**

library(vegan)

rowdata<-read.delim("test.txt",row.names = 1,header = T)

dis_bray<- vegdist(rowdata,method="bray")

clust_single <- hclust(dis_bray, method = 'complete')

plot(clust_single,hang = -1, cex = 0.6)

library(dendextend)

color<-read.csv("color.csv",header = T)

color<-as.vector.factor(color$G)

color<-c(color)

dend <- as.dendrogram(clust_single)

dend %>% set("leaves_pch", 19) %>% # node point type

set("leaves_cex", 1.0) %>% # node point size

set("leaves_col", color) %>% #node point color

set("labels_cex", 0.6)%>%

plot(main = "Leaves points",cex = 0.4)

**AUCRF**

mydata<- read.csv("RF2.csv", header = T, row.names = 1)

str(mydata)

mydata$Group<- as.factor(mydata$Group)

library(AUCRF)

set.seed(03)

mymodel<- AUCRF(Group~.,data=mydata)

mymodelcv<- AUCRFcv(mymodel,nCV=10,M=10)

summary(mymodelcv)

plot(mymodelcv)

attributes(mymodelcv)

**RF model construction with cross-validation**

mydata<- read.csv("1_2_TOP4.csv", header = T,row.names = 1)

str(mydata)

mydata$Group<- as.factor(mydata$Group)

library(caret)

library(randomForest)

set.seed(04)

splitRule<- trainControl(method = "repeatedcv", number = 10,repeats = 10,

classProbs = T, summaryFunction =twoClassSummary)

model <- train(

Group ~., data = mydata, method = "rf",

trControl = splitRule,

importance = TRUE,metric="ROC")

model$bestTune

modelf<-model$finalModel

modelf

importance(modelf)

attributes(modelf)

predicted.classes <- predict(modelf, newdata=mydata,type="prob")

predicted.classes

varImpPlot(model$finalModel, type = 2)

varImp(model)

**RF model test**

testdata<-read.csv("1_2_TOP4.csv", header = T,row.names = 1)

testdata$Group<- as.factor(testdata$Group)

predicted.classes <- predict(modelf, newdata=testdata,type="prob")

predicted.classes
