# Supplementary material for: Gut Microbiota-Based Algorithms in the Prediction of Metachronous Adenoma in Colorectal Cancer Patients Following Surgery
Source: Front Microbiol. 2020 Jun 12;11:1106. doi: 10.3389/fmicb.2020.01106 (PMC7303296; doi:10.3389/fmicb.2020.01106)
Supplement: Supplementary file 4 [file Table_2.docx]

**Table S2. Clinico-pathological Characteristics of Patients.**

|  | **MA (n=21)** | | | **nMA (n=24)** | | **P-value** | |
| --- | --- | --- | --- | --- | --- | --- | --- |
| **Gender** |  | | |  | |  | |
| Female | 8 | | | 8 | | 0.739 | |
| Male | 13 | | | 16 | |  | |
| **Age (years)^a^** | 65 (59.25-69.25) | | | 66 (52.5-68) | | 0.707 | |
| **BMI^a^** | 25.39 (23.31-26.73) | | | 22.3 (20.21-24.34) | | 0.005^*^ | |
| **Synchronous adenoma** |  | | |  | |  | |
| Yes | 15 | | | 10 | | 0.045^*^ | |
| No | 6 | | | 14 | |  | |
| **Bowel**  **obstruction^d^** |  | | |  | |  | |
| Yes | 3 | | | 4 | | 0.826 | |
| No | 18 | | | 20 | |  | |
| **Hematochezia** |  | | |  | |  | |
| Yes | 8 | | | 13 | | 0.281 | |
| No | 13 | | | 11 | |  | |
| **Tumor size^ac^** | 4 (4-5) | | | 4 (3-5) | | 0.602 | |
| **Tumor location^b^** |  | | |  | |  | |
| Left hemi-colon | 2 | | | 4 | | 0.770 | |
| Right hemi-colon | 8 | | | 8 | |  | |
| Rectum | 11 | | | 12 | |  | |
| **CEA^a^** | 3.07 (1.67-11.94) | | | 5 (3.25-12.74) | | 0.195 | |
| **CA 19-9^a^** | 11.8 (7.79-29.28) | | | 15.23 (8.15-35.775) | | 0.682 | |
| **Adjuvant therapy** |  | | |  | |  | |
| Yes | | 14 | 15 | | 0.771 | |  |
| No | 7 | | | 9 | |  | |
| **TNM-Stage** |  | | |  | |  | |
| I | 2 | | | 1 | | 0.95 | |
| IIA | 9 | | | 14 | |  | |
| IIIA | 1 | | | 0 | |  | |
| IIIB | 9 | | | 7 | |  | |
| IIIC | 0 | | | 2 | |  | |

^*^P<0.05, different from controls by Wilcoxon rank-sum test or Chi-squared test for continuous or categorical variables, respectively.

^a^ Data shown as median (1st and 3rd quartile).

^b^Tumor location: splenic flexure, descending, sigmoid, rectosigmoid were classified as left hemi-colon; ileocecal, ascending, hepatic flexure, transverse were classified as right hemi-colon.

^c^Tumor size defination: maximum diameter.

^d^Bowel obstruction was defined when coloscopy cannot pass through the tumor obstruction.

CEA: carcinoembryonic antigen.

CA19-9: carbohydrate antigen 19-9.
