# Supplementary material for: Gut Microbiota-Based Algorithms in the Prediction of Metachronous Adenoma in Colorectal Cancer Patients Following Surgery
Source: Front Microbiol. 2020 Jun 12;11:1106. doi: 10.3389/fmicb.2020.01106 (PMC7303296; doi:10.3389/fmicb.2020.01106)
Supplement: Supplementary file 5 [file Table_3.docx]

**Table S3. Clinico-pathological Characteristics of Patients.**

|  | **MA (n=11)** | | | **nMA (n=8)** | | **P-value** | |
| --- | --- | --- | --- | --- | --- | --- | --- |
| **Gender** |  | | |  | |  | |
| Female | 6 | | | 2 | | 0.352 | |
| Male | 5 | | | 6 | |  | |
| **Age (years)^a^** | 67.8 (63-72.5) | | | 54.5 (49.75-58.75) | | 0.001^*^ | |
| **BMI^a^** | 25 (22.4-26.05) | | | 23.7 (21.72-24.2) | | 0.432 | |
| **Synchronous adenoma** |  | | |  | |  | |
| Yes | 7 | | | 3 | | 0.370 | |
| No | 4 | | | 5 | |  | |
| **Bowel**  **obstruction^d^** |  | | |  | |  | |
| Yes | 0 | | | 1 | | 0.421 | |
| No | 11 | | | 7 | |  | |
| **Hematochezia** |  | | |  | |  | |
| Yes | 7 | | | 6 | | 1 | |
| No | 4 | | | 2 | |  | |
| **Tumor size^ac^** | 4.3 (3.75-5.75) | | | 4 (3.35-5) | | 0.296 | |
| **Tumor location^b^** |  | | |  | |  | |
| Left hemi-colon | 5 | | | 0 | | 0.158 | |
| Right hemi-colon | 1 | | | 3 | |  | |
| Rectum | 5 | | | 5 | |  | |
| **CEA^a^** | 5.89 (3.465-12.185) | | | 7.38 (4.1-21.14) | | 0.409 | |
| **CA 19-9^a^** | 9.8 (7.125-17.56) | | | 14.92 (10.435-85.173) | | 0.322 | |
| **Adjuvant therapy** |  | | |  | |  | |
| Yes | | 6 | 6 | | 0.633 | |  |
| No | 5 | | | 2 | |  | |
| **TNM-Stage** |  | | |  | |  | |
| I | 1 | | | 0 | | 0.421 | |
| IIA | 6 | | | 3 | |  | |
| IIIA | 0 | | | 0 | |  | |
| IIIB | 4 | | | 3 | |  | |
| IIIC | 0 | | | 2 | |  | |

^*^P<0.05, different from controls by Wilcoxon rank-sum test or Chi-squared test for continuous or categorical variables, respectively.

^a^ Data shown as median (1st and 3rd quartile).

^b^Tumor location: splenic flexure, descending, sigmoid, rectosigmoid were classified as left hemi-colon; ileocecal, ascending, hepatic flexure, transverse were classified as right hemi-colon.

^c^Tumor size definition: maximum diameter.

^d^Bowel obstruction was defined when coloscopy cannot pass through the tumor obstruction.

CEA: carcinoembryonic antigen.

CA19-9: carbohydrate antigen 19-9.
